# Internet Information on Oral Cancer Drugs: a Critical Comparison between Website Providers

**DOI:** 10.1007/s13187-020-01909-9

**Published:** 2020-10-30

**Authors:** Berit Bartmann, Henriette Schallock, Clara Dubois, Christian Keinki, Bijan Zomorodbakhsch, Michael Hartmann, Jutta Hübner

**Affiliations:** 1 Medizinische Klinik II, Hämatologie und Internistische Onkologie, Universitätsklinikum Jena, Am Klinikum 1, 07747 Jena, Germany; 2 MVZ Onkologische Kooperation Harz GbR, Goslar, Germany; 3 Klinikumsapotheke, Universitätsklinikum Jena, Jena, Germany

**Keywords:** Internet, Information quality, Transparency, E-health literacy, Oral cancer drugs

## Abstract

Cancer patients need access to high-quality information, when making decisions about oral cancer drugs. The internet is often used as a source of information published by highly heterogeneous providers. The objective was to evaluate the quality of website providers supplying online information about oral cancer drugs. One hundred websites were analyzed using content-related and formal criteria, selected from three existing evaluation methods used for cancer websites, for medical information (defined by the German Agency for Quality in Medicine), and for the “fact box” tool. A web search by a patient was simulated to identify websites to evaluate. ANOVA was used to assess information provided by non-profit organizations (governmental and non-governmental), online newspapers, for-profit organizations, and private/unknown providers. Content-related quality differences were found between online newspapers and all other categories, with online newspapers ranking significantly lower than for-profit and non-profit websites. As for formal criteria, for-profit providers scored significantly lower than non-profit providers and online newspapers for the aspect of transparency. Internet information on oral cancer drugs published by non-profit organizations constitutes the best available web-based source of information for cancer patients. Health literacy and e-health literacy should be promoted in the public domain to allow patients to reliably apply web-based information. Certification should be required by law to ensure fulfillment of requirements for data reliability and transparency (authorship and funding) before health professionals recommend websites to cancer patients.

**Supplementary Information:**

The online version contains supplementary material available at 10.1007/s13187-020-01909-9.

## Introduction

In modern medicine, patients want to be involved in decisions about their medical treatment. One aim is to support the patient’s right to take their own decisions and respect their individuality [1]. Evidence exists that such patient-centered communication and shared decision-making may lead to more realistic expectations about treatment goals, increased quality of life, and therefore, higher adherence [2–4].

Oral chemotherapies are used primarily in the outpatient setting. Compared to intravenous therapies, oral chemotherapy adherence is much more demanding for both patients and physicians. Physicians are not in direct control of the correct drug intake. Patients must remember to take their medication even when feeling unwell or fearing possible side effects. Moreover, with patients taking their drugs for long periods without direct interaction with their oncologist, few opportunities arise for patients to seek and receive reassurance, for example about precautions or adverse reactions. With the growing number of oral cancer drugs, adherence is becoming a central topic of concern in cancer care. For the reasons described above, it is strongly believed that adherence may be higher for well-informed patients and after shared decision-making [2, 5]. One decisive prerequisite is that patients have access to high-quality information [6].

One information source is the medication package leaflet, which pharmaceutical manufacturers are obliged to provide with detailed information on each drug. Yet, Pires and colleagues have shown that package leaflets cause severe readability problems, due to complex and difficult phrasing and small font size [7]. Consequently, patients search for other information sources. The internet is easily accessed and is often used to search for medical information [8, 9]. According to Hämeen-Anttila and colleagues, the internet is one of the most common sources of information about medication, following the package leaflet, and consultation of health professionals, such as pharmacists and physicians [8]. The study by Shea-Budgell and colleagues suggests that the internet is not only one of the most common sources but also one of the most trusted [10]. This contrasts sharply with the quality of websites that are usually used by cancer patients. Liebl and colleagues demonstrated that high-quality websites cannot be easily found by patients. In their study, most high-quality websites had low visibility [11]. In addition, cancer websites reviewed by Haase and colleagues were not as comprehensive as they should be. According to Haase, websites provide information in a one-sided way. Moreover, websites claimed their information was correct without quoting scientific references [12].

Since most patients have no scientific education and e-health literacy tends to be low [13], it is difficult for them to decide which websites provide evidence-based, qualified information. Thus, it might be helpful for health professionals to recommend relevant websites to patients. To do this, they need to know which providers supply qualified information. In 2015, Liebl and colleagues analyzed cancer-related websites in order to rate provider quality [11]. They evaluated the websites in terms of content-related and formal criteria. In their analysis, the best overall score and the best formal score were achieved by websites hosted by statutory health insurance and non-profit organizations.

Herth and colleagues analyzed internet information for patients on cancer diets [14]. In their analysis, self-help groups and non-profit organizations ranked highest. By contrast, for-profit groups, newspapers, and websites of individual physicians ranked lowest. In both studies [11, 14], the quality of non-profit websites was higher than the quality of websites provided by the for-profit sector and online newspapers. However, it is not clear if these findings can be applied to other cancer topics, such as oral cancer drugs.

To our knowledge, no structured analysis regarding information on oral cancer drugs has been published to date. Therefore, the primary focus of this paper was to assess the quality of websites on oral cancer drugs and to evaluate differences between website providers concerning content-related and formal information quality.

## Materials and Methods

### Assessment Instrument

The aim of our study was to assess the quality of web-based information on cancer drugs targeted at laypeople by developing an instrument for systematically analyzing oral cancer drug websites. Since the instrument developed by Liebl et al. [11] has been tried and tested for analysis of cancer websites in general, we used it as a basis for our instrument. Next, we added drug-specific criteria, derived from a literature search on scientifically established quality criteria. The original Liebl instrument consists of 18 content-related aspects and 6 formal aspects, each comprising several assessment criteria. It is based on criteria from:

- the HONcode (Health On the Net Foundation Code of Conduct) [15], the DISCERN [16] instrument, and the Germany-based AFGIS certification [17] (Aktionsforum Gesundheitsinformationssystem; − the “Health Information System Action Forum”), all of which define quality and transparency criteria for medical websites,
- the guidelines published by the ÄZQ (Ärztliches Zentrum für Qualität in der Medizin - a Germany-based agency for medical quality) [18], and
- the publication from Steckelberg and colleagues from the German “Network for Evidence-Based Medicine” (EBM-Network) [19], which defines how to present current evidence-based information in a way that is comprehensive and understandable for patients.

For our instrument, we reviewed and integrated criteria from the ÄZQ guidelines to identify criteria relevant for medication information [18] and added drug-specific criteria, derived from a literature search on scientifically established quality criteria. Additionally, we derived criteria from the article by Gigerenzer and colleagues [20], who developed the “fact box” tool, which is used to present evidence-based information in a clearly structured and easy to understand format [20].

We aggregated these sources to create an inventory of content-related and formal criteria to assess.

Finally, our instrument (Table 1) consisted of eight content-related and six formal aspects, each determined by criteria from the inventory. All individual quality criteria were assessed separately by two of the authors and summarized in one overall score for each aspect.

**Table 1:** Instrument consisting of content-related and formal criteria

Type of aspect	Aspect	Criteria	ICC^a^ (95% CI^b^)
Content-related	Summarized quality of information of whole website	•Is it easy to understand? •Are the objectives and target audience clear and does it achieve those objectives? •Is it written neutrally and fairly balanced? •Does it focus on the patient? •Are layout aspects taken into consideration? •Is information clearly arranged and is there a search function? •Is missing evidence communicated clearly?	0.735 (0.583–0.828)
	Discrepancies from package leaflet	•Is the wording of the package leaflet used?	
	Suitability to support shared decision-making	•Does the information on indication and usage support shared decision-making? •Does the information on contraindications and warnings support shared decision-making? •Does the information on precautions support shared decision-making? •Does the information on adverse reactions support shared decision-making? •Does the further information support shared decision-making?	0.838 (0.745–0.895)
	Quality of information on indications and usage	•Does the data apply to endpoints and are objective results presented first, followed by subjective results? •Are inter-individual differences considered? •Was the medication compared to a placebo and is it presented in a way that makes the effect clear? •Does it describe the benefit of the medication? •Does it describe the mode of action? •Does it describe the risks? •Does it describe the consequences of non-treatment?	0.909 (0.865–0.939)
	Quality of information on contraindications and warnings	•Are the information and the context relevant to the individual? •Are inter-individual differences considered? •Is the data based on current scientific evidence? •Are there no statements on topics without evidence?	0.849 (0.637–0.923)
	Quality of information on precautions	•Are the statements precise? •Are inter-individual differences considered? •Is the data based on current scientific evidence? •Are there no statements on topics without evidence?	0.807 (0.712–0.870)
	Quality of information on adverse reactions	•Does the data apply to endpoints and are objective results presented first, followed by subjective results? •Are the risks presented in a way that helps to weigh up the risks and benefits? •Is it presented in suitable graphics that clearly present the effect, in absolute frequencies and always with the same form of expression? •Is it based on current scientific evidence? •Are there no statements on topics without evidence?	0.607 (0.335–0.758)
	Quality of further information	•Is the information focused on the patient? •Are the statements precise? •Are inter-individual differences considered? •Is the data based on current scientific evidence? •Are there no statements on topics without evidence?	0.803 (0.708–0.867)
Formal	Transparency	•Are the authors and source of information given? •Is there any information about the provider? •Is the funding communicated clearly? •Is the advertisement policy communicated clearly, the advertisement clearly labeled as advertisement and clearly separated from information? •Are sponsors and partners listed?	0.850 (0.764–0.903)
	Privacy protection	•Is there information about privacy protection?	
	Completeness of information on sources of evidence	•Are the sources of evidence clearly communicated? •Is it clearly communicated when the information was written?	
	Observance of scientific standards and conventions on the presentation of numbers and outcomes	•Is observance of scientific standards and conventions on the presentation of numbers and outcomes taken into consideration?	
	Language adapted to the needs of the target group	•Is the language adapted to the needs of the target group? Is the language adapted to the needs of the target group?	
	Options for user feedback and participation	•Is there a possibility for users to give feedback?	

^a^Inter-class correlation

^b^Confidence interval

### Content-Related Quality Aspects

For content-related quality, we applied three general aspects to the entire website. Five aspects refer to the package leaflet structure officially defined by the EMA (European Medicines Agency) and by the AMG (the German Medicinal Product Act – Arzneimittelgesetz). To each of those aspects, we assigned various criteria from the instruments described above that determine the information quality. These criteria were subsequently summarized into one overall score per aspect. To structure the assessment of content-related quality, we used the package leaflet structure that is officially defined by the AMG.

Consequently, the content-related website analysis of this study consists of eight aspects. On the one hand, three aspects relate to the whole website: summarized quality of information of the whole website, discrepancies from the package leaflet, and suitability to support shared decision-making. On the other hand, five aspects relate to the information quality of topics covered by the structure of the package leaflets: indications and usage, contraindications and warnings, precautions, adverse reactions, and further information.

To each of those aspects, we assigned various criteria from the instruments described above. Some criteria were relevant for every aspect, such as “scientific evidence and timeliness” (“Is the data based on current scientific evidence?”) and “no statements on topics without evidence” (“Are there no statements on topics without evidence?”), and were therefore assigned to all aspects. Others, such as “risk communication” (“Are the risks presented in a way that helps to weigh up the risks and benefits?”) and “intelligibility for laypeople” (“Is it presented in suitable graphics that clearly present the effect, in absolute frequencies and always with the same form of expression?”) were only assigned to the relevant aspects, such as “quality of information on adverse reactions.” For example, the aspect “quality of information on precautions” contains 4 individual quality criteria: “rigor” (“Are the statements precise?”), “consideration of inter-individual differences” (“Are inter-individual differences considered?”), “scientific evidence and timeliness” (“Is the data based on current scientific evidence?”), and “no statements on topics without evidence” (“Are there no statements on topics without evidence?”). A summarized score for the four criteria was calculated to receive an overall quality measure for the aspect “quality of information on precautions.” The whole instrument can be seen in Table 1.

### Formal Quality Aspects

In addition to the eight content-related aspects, we evaluated six formal aspects. Once again, the aspects consist of individual criteria. For example, the aspect “transparency” consists of five criteria: “transparency concerning authors and source” (“Are the authors and source of information given?”), “transparency concerning provider” (“Is there information about the provider?”), “transparency concerning funding” (“Is the funding communicated clearly?”), “transparency concerning advertisement” (“Is the advertisement policy communicated clearly, the advertisement clearly labeled as advertisement and clearly separated from information?”), and “transparency concerning supporters” (“Are sponsors and partners listed?”). The resulting instrument can be seen in Table 1.

### Inter-Rater Reliability

To assess inter-rater reliability, eight sub-totals were built: one for each of the different content-related aspects and one for all formal aspects together. Inter-rater reliability was analyzed using inter-class correlation [21].

### Selection of Websites to Analyze

To identify relevant website articles published in German, we simulated an internet search of patients looking for information on their oral cancer drugs. Since the focus was on information websites, forums were excluded. To achieve the most objective results, we deleted cookies and used three different search engines: Google, Yahoo, and Bing. As a search term we used “<<Brand name>>” (“<<Handelsname>>”). We added “side effects” (“Nebenwirkungen”) to find websites that contained more detailed information about the drug and may therefore be used more often by patients requiring a large amount of information to give them the ability and confidence to make good decisions about their therapy. Pages advertised by search engines were excluded. The search was conducted for 10 different oral cancer drugs in June 2018: Capecitabine (Xeloda®), Cyclophosphamid (Endoxan®), Hydroxycarbamid (Litalir®), Tamoxifencitrat (Nolvadex®), Bicalutamid (Casodex®), Osimertinib (Tagrisso®), Ceritinib (Zykadia®), Ibrutinib (Imbruvica®), Dabrafenib (Tafinlar®), Crizotinib (Xalkori®).

We analyzed the first 10 search results for each drug. Websites providing articles about more than one drug were analyzed for each drug, each time focusing on the respective article. So, the content-related quality assessment focused on the individual article about the drug. We defined 4 categories of websites. Category 1 contains websites run by non-profit organizations (governmental and non-governmental), professional associations (non-profit), health project groups, and self-help groups. Category 2 relates to websites with a journalistic background, such as online newspapers and magazines (for the purposes of the assessment, these are jointly referred to as “online newspapers”). Category 3 consists of online pharmacies, pharmaceutical companies, and other for-profit organizations. Category 4 includes websites hosted by private persons and websites where no information about the provider was clearly visible.

### Assessment of Websites

All websites were assessed between June and August 2018. Two of the authors independently evaluated all selected websites using the instrument described above (Table 1). The rating was done using a 3-level Likert scale: “0 = the website violates the criterion at several points or in decisive elements, 1 = the website is partly in accordance with the criterion, yet there are some drawbacks, 2 = the website is in full accordance with the criterion” [11]. In consequence, the points achievable ranged from 0 (low) to 74 (high) for the 37 content-related quality criteria, and from 0 (low) to 22 (high) for the 11 formal quality criteria. To obtain the final quality scores, the arithmetic means of the ratings given by the two raters were calculated for each criterion. Subsequently, the criteria mean for each content-related aspect was summarized, resulting in seven content-related scores, and the criteria mean for each formal aspect was summarized, resulting in six formal scores. In addition, one overall quality score combining all content-related and formal aspects, except for the “discrepancies from package leaflet,” was defined. As a result, 14 scores were defined for comparison.

### Statistical Analyses

To test whether there was a significant difference in quality between the four categories of website providers, Welch tests were calculated including post hoc procedures. Levene’s test showed that the assumption of homogeneity of variance was violated for several quality aspects. Therefore, the Welch test was used as a robust ANOVA method. To avoid a type I error caused by different sample sizes or variances of homogeneity, we also used a robust post hoc procedure: The Games-Howell procedure. The Welch test was used for several comparisons. First, the overall quality score was compared between the four website categories. For this purpose, the mean overall quality scores of each website category were compared. Secondly, additional Welch tests were run for each quality aspect to assess the specific aspects, which differed between the four website categories. To determine which groups differed exactly, the Games-Howell test was run. All analyses were conducted using IBM SPSS 24 for Windows.

## Results

We analyzed 100 website articles published by 39 website providers. 23 articles were provided by the non-profit sector, 14 by online newspapers, 57 by online pharmacies, pharmaceutical firms, and other for-profit providers, and 6 articles by private persons or where no information was included about the provider (Table 2). The inter-class correlation for every sum ranged from 0.607 to 0.909 (Table 1). The inter-class correlation for the aspect “quality of information on adverse reactions” was moderate, the inter-class correlation for “quality of information on indication and usage” was excellent, and all other inter-class correlations were good [21].

**Table 2:** Quality scores of websites

Drug	Website	Category	Overall quality score 0 (low) to 96 (high)	Content-related score 0 (low) to 74 (high)	Formal score 0 (low) to 22 (high)
Capecitabine (Xeloda®)	www.onmeda.de	for-profit	83.83	66.33	17.50
	www.sanego.de	For-profit	80.58	65.58	15.00
	www.ema.europa.eu	Non-profit	76.17	61.17	15.00
	www.sanicare.de	For-profit	75.17	62.17	13.00
	medikamio.com	For-profit	73.00	68.50	4.50
	de.wikipedia.org	Non-profit	65.58	48.58	17.00
	www.bcaction.de	Non-profit	60.33	39.33	21.00
	flexikon.doccheck.com	For-profit	55.33	43.33	12.00
	www.apotheken-umschau.de	Online newspaper	42.00	30.00	12.00
	www.nebenwirkungen.co	For-profit	34.58	28.58	6.00
Cyclo-phosphamid (Endoxan®)	www.onmeda.de	For-profit	82.08	64.08	18.00
	www.compendium.ch	For-profit	78.08	66.08	12.00
	imedikament.de	For-profit	75.83	68.33	7.50
	www.medikamente-per-klick.de	For-profit	74.25	65.25	9.00
	www.netdoktor.de	For-profit	71.83	57.33	14.50
	de.wikipedia.org	Non-profit	69.08	52.08	17.00
	www.success-studie.de	Non-profit	68.58	58.58	10.00
	medikamio.com	For-profit	65.33	61.83	3.50
	www.gifte.de	Private/unknown	63.58	57.08	6.50
	www.apotheken-umschau.de	Online newspaper	42.00	30.00	12.00
Hydroxycarbamid (Litalir®)	www.medikamente-per-klick.de	For-profit	78.50	65.00	13.50
	www.onmeda.de	For-profit	76.58	58.58	18.00
	www.compendium.ch	For-profit	74.75	62.75	12.00
	www.gelbe-liste.de	For-profit	74.08	63.08	11.00
	de.wikipedia.org	Non-profit	72.42	55.92	16.50
	medikamio.com	For-profit	70.25	65.75	4.50
	www.chemie.de	For-profit	67.92	57.42	10.50
	imedikament.de	For-profit	67.25	61.25	6.00
	www.ellviva.de	For-profit	61.75	53.25	8.50
	www.apotheken-umschau.de	Online newspaper	42.00	29.50	12.50
Tamoxifencitrat (Nolvadex®)	www.netdoktor.de	for-profit	85.08	69.08	16.00
	www.onmeda.de	For-profit	79.42	61.42	18.00
	de.wikipedia.org	Non-profit	78.92	60.92	18.00
	www.gesundheit.de	For-profit	72.67	62.17	10.50
	www.compendium.ch	For-profit	72.17	60.17	12.00
	www.lifeline.de	For-profit	72.00	60.50	11.50
	www.biokrebs.de	Non-profit	67.08	53.58	13.50
	www.apotheken-umschau.de	Online newspaper	41.33	28.83	12.50
	www.nebenwirkungen.co	For-profit	32.67	26.67	6.00
	de.thinksteroids.com	For-profit	30.17	26.17	4.00
Bicalutamid (Casodex®)	www.netdoktor.de	For-profit	82.75	67.75	15.00
	prostatakrebs-tipps.de	Private/unknown	77.67	64.17	13.50
	www.compendium.ch	For-profit	76.92	64.92	12.00
	www.onmeda.de	For-profit	75.42	56.92	18.50
	medikamio.com	For-profit	69.50	64.00	5.50
	www.ellviva.de	For-profit	65.83	56.33	9.50
	www.prostatakrebse.de	Non-profit	56.42	43.42	13.00
	www.arznei-telegramm.de	Online newspaper	53.08	38.58	14.50
	www.apotheken-umschau.de	Online newspaper	41.33	29.33	12.00
	www.nebenwirkungen.co	For-profit	32.17	26.17	6.00
Osimertinib (Tagrisso®)	www.gesundheitsinformation.de	Non-profit	82.58	62.08	20.50
	www.gelbe-liste.de	For-profit	81.33	70.33	11.00
	www.onmeda.de	For-profit	80.92	63.42	17.50
	www.ema.europa.eu	Non-profit	76.75	61.25	15.50
	www.pharmazeutische-zeitung.de	Online newspaper	73.42	58.42	15.00
	www.oncotrends.de	Private/unknown	70.75	59.25	11.50
	medikamio.com	For-profit	68.75	65.25	3.50
	imedikament.de	For-profit	65.50	57.50	8.00
	arznei-news.de	For-profit	60.08	45.58	14.50
	www.tabletwise.com	For-profit	53.92	47.92	6.00
Ceritinib (Zykadia®)	www.ema.europa.eu	Non-profit	85.75	70.25	15.50
	www.onmeda.de	For-profit	84.67	67.17	17.50
	www.compendium.ch	For-profit	83.92	71.92	12.00
	de.wikipedia.org	Non-profit	83.17	63.17	20.00
	www.gelbe-liste.de	For-profit	79.67	69.67	10.00
	www.medihelp.ch	Private/unknown	73.83	68.83	5.00
	imedikament.de	For-profit	69.00	61.00	8.00
	www.allmedicines.net	Private/unknown	64.58	55.58	9.00
	arznei-news.de	For-profit	60.83	48.83	12.00
	www.apotheken-umschau.de	Online newspaper	41.33	28.83	12.50
Ibrutinib (Imbruvica®)	www.iwmf.com	Non-profit	88.67	72.17	16.50
	ec.europa.eu	Non-profit	83.58	72.58	11.00
	www.ema.europa.eu	Non-profit	81.50	67.00	14.50
	www.pharmazeutische-zeitung.de	Online newspaper	80.92	67.92	13.00
	www.compendium.ch	For-profit	78.83	66.83	12.00
	imedikament.de	For-profit	74.50	67.50	7.00
	arznei-news.de	For-profit	68.17	55.67	12.50
	www.krebsgesellschaft.de	Non-profit	66.17	47.67	18.50
	www.tabletwise.com	For-profit	53.42	47.92	5.50
	www.apotheken-umschau.de	Online newspaper	41.33	29.33	12.00
Dabrafenib (Tafinlar®)	www.gesundheitsinformation.de	Non-profit	81.17	61.67	19.50
	www.pharmazeutische-zeitung.de	Online newspaper	80.67	64.67	16.00
	www.compendium.ch	For-profit	79.08	67.08	12.00
	www.onmeda.de	For-profit	78.67	61.17	17.50
	www.ema.europa.eu	Non-profit	73.50	59.00	14.50
	imedikament.de	For-profit	68.17	60.67	7.50
	arznei-news.de	For-profit	63.17	49.67	13.50
	www.gesundheitsstadt-berlin.de	Non-profit	61.00	46.50	14.50
	www.oncotrends.de	Private/unknown	55.75	44.25	11.50
	www.apotheken-umschau.de	Online newspaper	41.33	28.83	12.50
Crizotinib (Xalkori®)	www.bfarm.de	Non-profit	86.58	70.08	16.50
	www.onmeda.de	For-profit	83.92	66.92	17.00
	www.gesundheitsinformation.de	Non-profit	81.42	62.42	19.00
	www.pfizer.de	For-profit	80.67	66.67	14.00
	www.pharmazeutische-zeitung.de	Online newspaper	74.92	60.42	14.50
	www.ema.europa.eu	Non-profit	74.58	60.08	14.50
	medikamio.com	For-profit	73.08	68.58	4.50
	arznei-news.de	For-profit	69.83	55.83	14.00
	www.apotheken-umschau.de	Online newspaper	41.33	28.83	12.50
	www.nebenwirkungen.co	For-profit	32.58	26.58	6.00

Eight articles fully and 44 articles partly used the package leaflet wording, compared to 48 articles, which did not. The highest score was achieved by www.iwmf.com, a non-profit organization, established and managed by patients. It achieved 88.67 out of 96 possible points based on its article about Ibrutinib (Imbruvica®). The lowest score with 32.17 out of 96 points was achieved by nebenwirkungen.co based on its article about Bicalutamid (Casodex®). “www.nebenwirkungen.co” is a for-profit website. All analyzed websites are listed in Table 2.

### Overall Quality

The overall quality scores differed significantly in statistical terms between the four website categories: Welch’s *F* (3, 20.82) 6.93, *p* = 0.002. The evaluation identified at least one significant difference between the individual website categories. The Games-Howell post hoc analysis revealed a significant difference on the one hand, between online newspapers and non-profit websites and on the other hand, between online newspapers and for-profit websites. Overall, online newspapers had a significantly lower overall quality score (mean (*M*) = 52.64, standard deviation (*SD*) = 16.69) than non-profit websites (− 22.18, 95% confidence interval (CI) [− 35.91, − 8.46], *M* = 74.83, *SD* = 9.16, *p* = 0.001) and for-profit websites (− 16.84, 95% CI [− 30.50, − 3.18], *M* = 69.48, *SD* = 13.91, *p* = 0.013). Furthermore, online newspapers scored lower than websites hosted by private or unknown providers (− 15.05, 95% CI [− 30.67, 0.57], *M* = 67.69, *SD* = 7.95, *p* = 0.061). No significant difference was identified in the overall quality score between any other categories.

### Content-Related Aspects

Similar results were found for each separate content-related aspect. For all content-related aspects, online newspapers had the lowest mean score (Table 3). The difference between non-profit, for-profit, and private/unknown providers was not significant for content-related aspects. The means of online newspapers were significantly lower for all quality aspects than the means of for-profit websites and they were also significantly lower than the means of non-profit websites (Table 3) for all quality aspects, with the exception of “quality of information on contraindications and warnings” and “quality of information on precautions.” So, for example the “quality of information on indication and usage” of online newspapers (*M* = 8.27, *SD* = 6.12) was significantly lower than that of non-profit providers (− 6.63, 95% CI [− 11.53, − 1.74], *M* = 14.90, *SD* = 2.24, *p* = 0.007), for-profit providers (− 5.32, 95% CI [− 10.21, − 0.42], *M* = 13.58, *SD* = 3.55, *p* = 0.031), and private/unknown providers (− 6.61, 95% CI [− 11.71, − 1.51], *M* = 14.88, *SD* = 1.80, *p* = 0.009).

**Table 3:** Content-related and formal quality scores of providers

Aspect	Category	Non-profit *M*^a^ *(SD*^b^*)*	Online newspapers *M*^a^ *(SD*^b^*)*	For-profit *M*^a^ *(SD*^b^*)*	Private/unknown *M*^a^ *(SD*^b^*)*	Welch test
Overall quality score		74.83 (9.16)	52.64 (16.69)	69.48 (13.91)	67.69 (7.95)	*F* (3, 20.82) = 6.93**, *p =* 0.002
Summarized quality of information of whole website		14.01 (2.29)	11.02 (2.14)	13.46 (2.52)	12.58 (2.79)	*F* (3, 18.97) = 5.69**, *p =* 0.006
Quality of information on indication and usage		14.90 (2.24)	8.27 (6.12)	13.58 (3.55)	14.88 (1.80)	*F* (3, 21.32) = 5.63**, *p* = 0.005
Quality of information on contraindications and warnings		5.02 (1.91)	4.16 (1.25)	6.15 (1.88)	5.38 (2.25)	*F* (3, 19.21) = 7.12**, *p* = 0.002
Quality of information on precautions		5.28 (1.82)	4.54 (1.08)	5.97 (1.71)	5.42 (1.43)	*F* (3, 20.18) = 4.82*, *p =* 0.011
Quality of information on adverse reactions		6.07 (0.95)	4.77 (0.82)	5.73 (0.97)	5.69 (0.81)	*F* (3, 19.75) = 6.58**, *p* = 0.003
Quality of further information		7.96 (1.08)	4.89 (1.98)	7.54 (1.81)	7.75 (0.99)	*F* (3, 20.93) = 8.99**, *p* = 0.001
Suitability to support shared decision-making		5.43 (2.32)	1.89 (2.90)	6.10 (2.49)	6.50 (2.41)	*F* (3, 18.87) = 8.07**, *p* = 0.001
Transparency		8.07 (1.34)	6.04 (0.84)	4.37 (2.82)	3.25 (1.72)	*F* (3, 21.14) =27.12**, *p* = 0.000
Privacy protection		1.93 (0.23)	1.68 (0.25)	1.82 (0.37)	1.00 (1.10)	*F* (3, 18.58) = 4.21*, *p* = 0.020
Completeness of information on sources of evidence		2.09 (1.46)	1.50 (0.65)	1.32 (0.95)	1.83(1.66)	*F* (3, 18.53) = 1.83, *p* = 0.183
Observance of scientific standards and conventions on the presentation of numbers and outcomes		1.46 (0.54)	1.29 (0.47)	1.14 (0.66)	1.50 (0.63)	*F* (3, 19.55) = 1.79, *p* = 0.183
Language adapted to the needs of the target group		1.09 (0.51)	0.96 (0.13)	1.03 (0.53)	1.00 (0.45)	*F* (3, 20.31) = 0.50, *p* = 0.688
Options for user feedback and participation		1.52 (0.46)	1.64 (0.23)	1.27 (0.66)	0.92 (0.80)	*F* (3, 20.07) = 4.74*, *p* = 0.012

^a^Mean

^b^Standard deviation

*p < 0.05

**p < 0.01

### Formal Aspects

Differences concerning formal aspects were more heterogeneous. The “transparency” score differed significantly between all categories (*F* (3, 21.14) = 27.11, *p* = 0.000). The highest mean was identified for non-profit websites (*M* = 8.07, *SD* = 1.34), which was significantly higher than all other categories, followed by (in descending order):

- online newspapers (− 2.03, 95% CI [− 3.00, − 1.06], *M* = 6.04, *SD* = 0.84, *p* = 0.000)
- for-profit websites (− 3.40, 95% CI [− 4.92, − 2.47], *M* = 4.37, *SD* = 2.81, *p* = 0.000)
- private/unknown websites (− 4.82, 95% CI [− 7.36, − 2.27], *M* = 3.25, *SD* = 1.72, *p* = 0.002).

A further significant difference was found for “privacy protection,” (*F* (3, 18.58) = 4.21, *p* = 0.020), where the mean of non-profit (*M* = 1.93, *SD* = 0.23) was significantly higher (*p* = 0.021) than the mean of online newspapers (*M* = 1.68, *SD* = 0.25) (0.26, 95% CI [0.03, 0.48]).

By contrast, online newspapers (*M* = 1.64, *SD* = 0.23) achieved the highest mean for the aspect “options for user feedback and participation,” which was significantly higher (*p* = 0.008) than the mean of for-profit providers (*M* = 1.27, *SD* = 0.66) (0.37, 95% CI [0.09, 0.66]).

The difference between categories for “completeness of information on sources of evidence,” “observance of scientific standards and conventions on the presentation of numbers and outcomes,” and “language adapted to the needs of the target group” was not significant (Table 3).

## Discussion, Limitations, and Conclusion

### Discussion

Our study shows that information quality differs significantly between different categories of providers. As we simulated a patient search using standard search engines, the analyzed websites can be considered representative for a general patient search. Based on previous studies, one might expect differences between the for-profit and non-profit sector [11, 14]. However, our study did not identify significant differences for most quality aspects between websites of for-profit and non-profit organizations. This contrasts with the results of the studies by Liebl and colleagues, and Herth and colleagues, which found significant differences regarding almost all quality aspects for for-profit and non-profit providers [11, 14]. In our study, online newspapers scored significantly lower on the overall score and on all content-related items. Therefore, a patient searching for comprehensive information on oral cancer drugs would be recommended not to use online newspapers as a primary source. The aim of online newspapers may not be to provide in-depth coverage of topics, but to inform their readers about the most recent developments. For example, “www.apotheken-umschau.de,” a website hosted by a popular German magazine about health, did not cover all sub-topics, such as indication and usage, and therefore did not provide comprehensive information. Since articles from online newspapers represent the smallest group analyzed, except from private and unknown providers, the question remains open whether analyzing more articles would lead to different results. This lower score for online newspapers is in line with the results of studies by Herth and colleagues, and Liebl and colleagues, where online newspapers also scored low for content-related quality [11, 14]. In terms of formal quality, the differences between providers were more heterogenous. For the privacy protection criteria, non-profit and for-profit providers did not differ significantly and reached a mean of over 90% of the achievable points. In the EU, privacy protection is controlled by the General Data Protection Regulation (GDPR) and non-compliance is severely sanctioned [22]. Consequently, providers abide by these rules. For criteria, which are not regulated by law, such as transparency, for-profit providers scored significantly lower than non-profit providers and online newspapers, obtaining fewer than half of the achievable points. Existing voluntary certifications, such as the HONcode, which aim to establish formal standards, for example, for transparency, are obviously insufficient to promote these qualities, most probably as they are not known to or not used by laypeople. Laversin and colleagues showed that most websites, which do not apply for HONcode certification, also do not fulfill its criteria [23]. A further website analysis conducted between April and July 2018 showed that privacy protection of websites increased after the GDPR reform in May 2018 [24]. One possibility for improving website quality may be certification required by law, to ensure fulfillment of requirements for data reliability, authorship, and funding. In times of rapidly changing online information, such a certification system may be hard to establish.

### Limitations

The aim of our study was to analyze websites that patients use to inform themselves about oral cancer drugs. Criticism may be raised that our simulated search is not representative of a real patient’s search. A future website analysis could repeat the search, asking real patients to run their own searches, and compare the results to the websites we found.

Some websites provided several articles on various cancer drugs. Thus, the websites were analyzed more than once, each time with a focus on another article. Accordingly, the quality of those websites has a higher weighting in our quality assessment than website providers with only one article.

### Conclusion

Patients taking oral cancer drugs have a high demand for quality sources of information about their drugs. Often, this source appears to be the internet. However, low health literacy and e-health literacy limit the patients’ ability to decide which websites are to be trusted. Our study showed quality differences between website providers. Yet, the content-related quality of non-profit and for-profit providers did not differ significantly, while the content-related quality of online newspapers was significantly lower. However, differences between non-profit and for-profit providers were identified regarding the aspect of transparency (including topics such as sources of information, providers, supporters, funding, and advertisement policy), with for-profit providers scoring significantly lower.

Some regulation concerning transparency seems mandatory. Yet, regulating the content-related quality of internet information may not be feasible. Therefore, oncologists should help their patients to find high-quality sources. In a first step, they should ask patients which sources of information they prefer or use. As a prerequisite, they should be familiar with high-quality websites for patients, and in a second step recommend non-profit websites.

Cancer associations should engage in providing an overview of high-quality websites for patients.

Besides, health literacy and e-health literacy are important skills for the health care system which must be promoted in a modern society. If we manage this in the whole population, patients will be able to refer to this knowledge and these skills in case of illness and will be in a position to navigate web-based information more easily and more reliably.

## Supplementary information

ESM 1 (DOCX 62 kb)

ESM 2 (DOCX 33 kb)

ESM 3 (DOCX 49 kb)

ESM 4 (DOCX 24 kb)
