# Supplementary material for: Internet Information on Oral Cancer Drugs: a Critical Comparison between Website Providers
Source: J Cancer Educ. 2020 Oct 30;37(4):983–93. doi: 10.1007/s13187-020-01909-9 (PMC9399062; doi:10.1007/s13187-020-01909-9)
Supplement: Supplementary file 1 — (DOCX 62 kb) [file 13187_2020_1909_MOESM1_ESM.docx]

**Figure S1: Quality aspects for assessment**

Notes: Q. o. i.: Quality of information
