# Supplementary material for: Internet Information on Oral Cancer Drugs: a Critical Comparison between Website Providers
Source: J Cancer Educ. 2020 Oct 30;37(4):983–93. doi: 10.1007/s13187-020-01909-9 (PMC9399062; doi:10.1007/s13187-020-01909-9)
Supplement: Supplementary file 2 — (DOCX 33 kb) [file 13187_2020_1909_MOESM2_ESM.docx]

**Figure S2: Mean of Overall Quality Score**

*p* = .296

*p* = .061

*p* = .001

*p* = .962

*p* = .194

*p* = .013

Notes: The significance of differences was measured using Games-Howell. There were significant differences found between online newspapers and non-profit and between online newspapers and for-profit websites (p < .05). No significant difference was found between the other categories (p > .05). Achievable points: 0 (low) to 96 (high).
