# Supplementary material for: Internet Information on Oral Cancer Drugs: a Critical Comparison between Website Providers
Source: J Cancer Educ. 2020 Oct 30;37(4):983–93. doi: 10.1007/s13187-020-01909-9 (PMC9399062; doi:10.1007/s13187-020-01909-9)
Supplement: Supplementary file 3 — (DOCX 49 kb) [file 13187_2020_1909_MOESM3_ESM.docx]

**Figure S3: Differences between transparency score of providers**

*p* = .002

*p* = .000

*p* =.002

*p* = .000

Notes: The significance of the differences of 'transparency' was measured using Games-Howell. The difference between each category was significant p < .05, except between for-profit websites and websites by private or unknown providers. Achievable points: 0 (low) to 10 (high)
