# Supplementary material for: Internet Information on Oral Cancer Drugs: a Critical Comparison between Website Providers
Source: J Cancer Educ. 2020 Oct 30;37(4):983–93. doi: 10.1007/s13187-020-01909-9 (PMC9399062; doi:10.1007/s13187-020-01909-9)
Supplement: Supplementary file 4 — (DOCX 24 kb) [file 13187_2020_1909_MOESM4_ESM.docx]

**Table S1 Quality differences between providers**

|  |  | **C^a^ 1 to C^a^ 2** | **C^a^1 to C^a^3** | **C^a^1 to C^a^4** | **C^a^2 to C^a^3** | **C^a^2 to C^a^4** | **C^a^3 to C^a^4** |
| --- | --- | --- | --- | --- | --- | --- | --- |
| Overview of q o i^b^ of whole article | *GH^c^* | -2.99** | -0.55 | -1.43 | 2.44** | 1.57 | -0.87 |
|  | *CI^d^* | -5.02  -0.97 | -2.11  1.00 | -5.53  2.68 | 0.61  4.27 | -2.55  5.69 | -5.00  3.26 |
|  | *P^e^* | .002 | .777 | .670 | .006 | .628 | .879 |
| Q o i^b^ on indication and usage | *GH^c^* | -6.63** | -1.32 | -0.03 | 5.32* | 6.61** | 1.29 |
|  | *CI^d^* | -11.53  -1.74 | -3.07  0.43 | -2.72  2.66 | 0.42  10.21 | 1.51  11.71 | -1.39  5.97 |
|  | *P^e^* | .007 | .203 | 1.000 | .031 | .009 | .484 |
| Q o i^b^ on contraindications and warnings | *GH^c^* | -0.86 | 1.13 | 0.35 | 1.99** | 1.21 | -0.77 |
|  | *CI^d^* | -2.26  0.54 | -0.13  2.39 | -2.95  3.66 | 0.85  3.12 | -2.10  4.53 | -4.11  2.56 |
|  | *P^e^* | .361 | .093 | .161 | .000 | .623 | .846 |
| Q o i^b^ on precautions | *GH^c^* | -0.75 | 0.69 | 0.13 | 1.44** | 0.88 | -0.56 |
|  | *CI^d^* | -2.03  0.54 | -0.50  1.88 | -2.01  2.27 | 0.44  2.44 | -1.23  2.99 | -2.66  1.55 |
|  | *P^e^* | .411 | .411 | .997 | .002 | .560 | .811 |
| Q o i^b^ on adverse reactions | *GH^c^* | -1.30** | -0.33 | -0.37 | 0.96** | 0.93 | -0.04, |
|  | *CI^d^* | -2.10  -0.50 | -0.97  0.30 | -1.58  0.84 | 0.26  1.66 | -0.30  1.15 | -1.23  1.16 |
|  | *P^e^* | .001 | .499 | .775 | .005 | .157 | 1.000 |
| Q of i^b^ on further information | *GH^c^* | -3.06** | -0.41 | -0.21 | 2.65** | 2.86 | 0.21 |
|  | *CI^d^* | -4.69  -1.44 | -1.28  0.45 | -1.67  1.26 | 1.01  4.29 | 0.97  4.75 | -1.26  1.67 |
|  | *P^e^* | .000 | .594 | .968 | .001 | .002 | .970 |
| Suitability to support shared decision-making    Notes: a Category, b quality of information, c Games-Howell, d 95%-confidence interval, e probability value  * p<0.05, ** p < 0.01 | *GH^c^* | -3.54** | 0.66 | 1.07 | 4.20** | 4.61* | 0.41 |
|  | *CI^d^* | -6.07  -1.01 | -0.90  2.23 | -2.49  4.62 | 1.82  6.59 | 0.86  8.35 | -3.15  3.96 |
|  | *P^e^* | .004 | .674 | .769 | .001 | .015 | .978 |
